# Effect of educational intervention for compliance of school adolescents with the human papillomavirus vaccine

**DOI:** 10.1590/1980-220X-REEUSP-2022-0082en

**Published:** 2022-10-07

**Authors:** Hellen Lívia Oliveira Catunda Ferreira, Cícero Mendes Siqueira, Leilane Barbosa de Sousa, Ana Izabel Oliveira Nicolau, Thaís Marques Lima, Priscila de Souza Aquino, Ana Karina Bezerra Pinheiro

**Affiliations:** 1 Universidade Federal do Ceará, Programa de Pós-Doutorado para Jovens Doutores da Fundação Cearense de Apoio ao Desenvolvimento Científico e Tecnológico. Fortaleza, CE, Brazil.; 2 Universidade Federal do Ceará, Departamento de Enfermagem, Fortaleza, CE, Brazil.; 3 Universidade da Integração Internacional da Lusofonia Afro-Brasileira, Instituto de Ciências da Saúde, Redenção, CE, Brazil.; 4 Universidade Federal do Ceará, Hospital Universitário Walter Cantídio, Fortaleza, CE, Brazil.; 5 Universidade Federal do Rio Grande do Norte, Faculdade de Ciências da Saúde do Trairi, Santa Cruz, RN, Brazil.; 6 Universidade Federal do Ceará, Programa de Pós-Graduação em Enfermagem, Fortaleza, CE, Brazil.

**Keywords:** Papillomavirus Vaccines, Clinical Trial, Educational Technology, Adolescent, School Health Services, Nursing, Vacunas contra Papillomavirus, Ensayo Clínico, Tecnología Educacional, Adolescente, Servicios de Salud Escolar, Enfermería, Vacinas contra Papilomavirus, Ensaio Clínico, Tecnología Educacional, Adolescente, Serviços de Saúde Escolar, Enfermagem

## Abstract

**Objective:**

to assess the effects of “Piss off, HPV!”, an educational intervention to increase adolescents’ knowledge, attitude and compliance with human papillomavirus vaccination.

**Method:**

a randomized clinical trial by cluster, carried out in six schools in two municipalities in Ceará, with 238 girls. The control group (n = 120) received routine instructions, and the intervention group (n = 118), printed message cards about the quadrivalent HPV vaccine. A pre- and post-intervention knowledge, attitude and practice survey was applied to both groups. The McNemar test, to analyze knowledge, attitude and pre- and post-intervention practice, the chi-square test, to compare compliance in relation to knowledge and attitude, and a logistic regression model, to assess vaccine compliance, were carried out. A significance level of 5% was adopted.

**Results:**

pre-intervention, knowledge was inadequate and attitude was adequate in both groups. Post-intervention, adequate knowledge and practices became greater in the intervention group. Adequate post-intervention knowledge and attitude, in addition to being 12 years of age or older, increase the chance for vaccination, explaining 70% of the practice.

**Conclusion:**

the educational intervention was effective for adolescents’ knowledge and compliance with the quadrivalent HPV vaccine. UTN: U1111-1254-5546; ReBEC: RBR-107hzdqt.

## RESUMO

**Objetivo:**

avaliar os efeitos da intervenção educativa “Sai fora, HPV!” para aumento do conhecimento, atitude e adesão de adolescentes à vacinação contra o papilomavírus humano.

**Método:**

ensaio clínico randomizado por conglomerado, realizado em seis escolas de dois municípios cearenses, com 238 meninas. O grupo controle (n = 120) recebeu orientações rotineiras, e o grupo intervenção (n = 118), cartões-mensagem impressos sobre a vacina HPV quadrivalente. Aplicou-se inquérito conhecimento, atitude e prática pré- e pós-intervenção em ambos os grupos. Realizaram-se Testes de McNemar, para analisar conhecimento, atitude e prática pré- e pós-intervenção, do Qui-Quadrado, para comparar adesão em relação ao conhecimento e atitude, e modelo de regressão logística, para avaliação da adesão à vacina. Adotou-se nível de significância 5%.

**Resultados:**

pré-intervenção, conhecimento era inadequado e atitude adequada em ambos os grupos. Pós-intervenção, conhecimento e práticas adequados se tornaram maior no grupo intervenção. Conhecimento e atitude adequados pós-intervenção, além da idade maior ou igual a 12, aumentam a chance para vacinação, explicando 70% da prática.

**Conclusão:**

a intervenção educativa foi efetiva para conhecimento e adesão da vacina HPV quadrivalente pelas adolescentes. UTN: U1111-1254-5546; ReBEC: RBR-107hzdqt.

## RESUMEN

**Objetivo:**

evaluar los efectos de la intervención educativa “¡Fuera VPH!” aumentar el conocimiento, la actitud y la adherencia de los adolescentes a la vacunación contra el virus papiloma humano.

**Método:**

ensayo clínico aleatorizado por conglomerados, realizado en seis escuelas de dos municipios de Ceará, con 238 niñas. El grupo de control (n = 120) recibió instrucciones de rutina y el grupo de intervención (n = 118), tarjetas con mensajes impresos sobre la vacuna tetravalente contra el VPH. A ambos grupos se les aplicó una encuesta de conocimientos, actitudes y prácticas pre y post intervención. Se realizaron las Pruebas de McNemar para analizar conocimientos, actitudes y prácticas pre y postintervención, Chi-Cuadrado, para comparar la adherencia en relación con conocimientos y actitudes, y un modelo de regresión logística para evaluar la adherencia a la vacuna. Se adoptó un nivel de significancia del 5%.

**Resultados:**

antes de la intervención, el conocimiento era inadecuado y la actitud adecuada en ambos grupos. Después de la intervención, el conocimiento y las prácticas adecuadas se hicieron mayores en el grupo de intervención. Un adecuado conocimiento y actitud post-intervención, además de tener 12 años o más, aumenta la posibilidad de vacunación, explicando el 70% de la práctica.

**Conclusión:**

la intervención educativa fue efectiva para el conocimiento y la adherencia de los adolescentes a la vacuna tetravalente contra el VPH. UTN: U1111-1254-5546; ReBEC: RBR-107hzdqt.

## INTRODUCTION

Human papillomavirus (HPV) is a sexually transmitted infection (STI) caused by a DNA virus in which its persistent cervical infection increases the risk of acquiring cancer and its precursor lesions^(1)^. The oncogenic subtypes that are most commonly associated with cervical cancer (CC) are subtypes 16 and 18^(2)^.

HPV vaccination represents an effective strategy for primary CC prevention, given its high efficacy against precancerous cervical lesions^(2)^. Globally, the highest prevalence of HPV is observed at young ages, peaking in women under 25 years of age, decreasing at older ages^(3)^.

In the European context, Infection rates and HPV incidence have dropped considerably since the vaccination license in 2006, which makes it possible to control the occurrence of cervical neoplasm^(4)^. Although the vaccine has been available in the Brazilian National Immunization Program (PNI – *Programa Nacional de Imunizações*) since 2014, it is difficult to maintain the expected vaccine coverage, especially for the second dose, weakening actions to combat cancer^(2)^.

Currently, the vaccine is administered in two doses, with an interval of 180 days between the first and second dose, in girls aged between nine and 14 years and in boys between 11 and 14 years old^(2)^.

A study that estimated the HPV 6, 11, 16, 18 (Recombinant) vaccine coverage in Brazil in adolescent girls in the year 2017 found that the first dose had high coverage in most cases, with the exception of the Federal District and Amazonas. However, for the second dose, the opposite was observed, with low vaccination coverage in all states^(5)^.

It appears that the main reasons for refusal occur due to lack of information, followed by psychogenic reaction, which is the set of symptoms that develop in response to stress associated with vaccination, such as fear of injection and adverse events, in addition to of finding that the vaccine was not effective^(6)^.

Thus, the use of attractive educational technologies that arouse curiosity about the subject and that are practical and easy to read is a form of learning support to enhance appropriate health practices aimed at vaccinating adolescents against HPV^(7)^.

It is noteworthy that the strategy based on printed message cards provides advantages, as it is an easily replicable, simple-to-use and low-cost technology compared to other types of technologies, being a palpable alternative for application, in addition to presenting adapted design and language, according to the target audience, with short and objective texts.

An American study used printed cards as an educational intervention to reduce the risk of STIs, concluding a decrease in risk behaviors (p < 0.001). Therefore, the data support the feasibility of providing a risk reduction intervention focused on card education, indicating that exposure to information on the subject may be associated with the acquisition of healthier practices^(8)^.

In this context, an educational health intervention through printed message cards, aimed at schoolchildren about HPV vaccination, can be a strategy for the development or reinforcement of capacities, in order to promote compliance with vaccination and continuity of the vaccination schedule, in addition to collaborating with care actions in Primary Health Care, focusing on adolescent health promotion and neoplasm prevention. The relevance of educational intervention as a health-promoting resource is highlighted, as it favors the action of adolescents as active agents of care. The relationship between health professionals and the target population, mediated by the message cards, enables knowledge promotion and attitudes towards healthy behaviors and adolescent health care.

In view of this, the objective is to assess the effects of “Piss off, HPV!” (*Sai fora, HPV*), through printed message cards to increase the knowledge, attitude and compliance of school adolescents with HPV vaccination.

It is noteworthy that the intervention named “Piss off, HPV” is part of an educational project linked to the Research Group on Sexual and Reproductive Health at the *Universidade Federal do Ceará* (UFC), with the purpose of promoting knowledge about HPV and its vaccine for adolescents and parents and/or guardians, in order to sensitize them about the importance and practice of immunization through playful activities.

## METHOD

### Study Design

This is an experimental study and a randomized clinical trial. There was a comparison between two groups: one with an educational intervention (IG), consisting of “Piss off, HPV!”, and educational project, with delivery of printed message cards about compliance with HPV vaccination for two consecutive months; and one with standard intervention, called control (CG), with only usual care, routine guidelines during nursing consultations at the health unit or guidelines passed on by the adolescent’s educational institution for the same period.

To ensure scientific rigor, the Consolidated Standards of Reporting Trials (CONSORT) guideline for non-pharmacological interventions was adopted as a methodological framework.

### Study Setting, Period and Population

The study took place in six elementary schools in two municipalities in the state of Ceará, northeastern Brazil, from August 2019 to January 2020. The study population consisted of female adolescents regularly enrolled in schools, which is justified due to the recent inclusion of male adolescents for HPV vaccination, in 2017.

### Selection Criteria and Sample Definition

**Figure 1.**
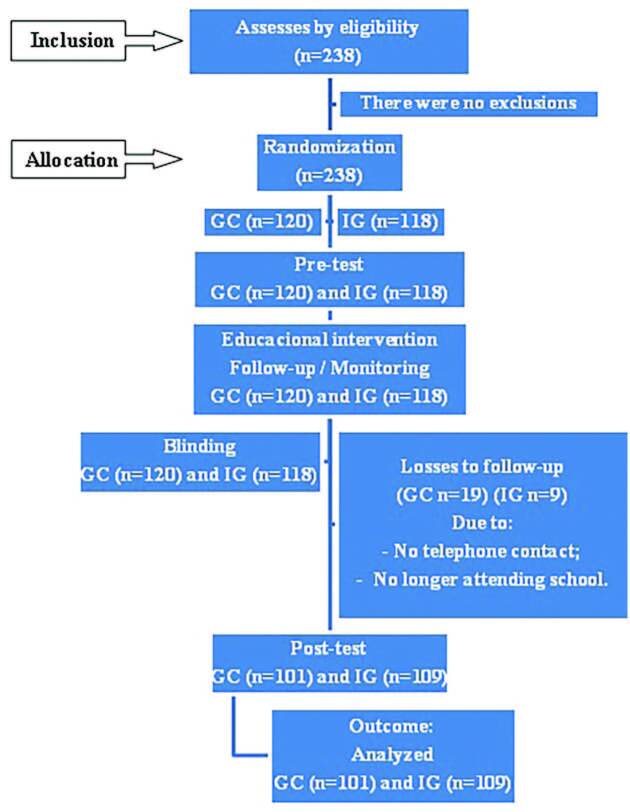
Representative diagram of participant flow at each moment of the study, according to the CONSORT guideline for non-pharmacological interventions – Fortaleza, CE, Brazil, 2022.

To calculate the sample, we used the formula for studies with comparative groups. With the HPV vaccination rate as the outcome variable, the following values were adopted: Zα = 95%, Zβ = 80%, p = 59.1%, d = 20%, with 95 participants per group. Adding a margin of 10% for possible losses, there were at least 105 adolescents in each group. Figure 1 shows the flowchart of participants at each moment of the study.

We included adolescent school girls aged 9 to 14 years who had received the quadrivalent HPV vaccine, attested by the vaccination card. We excluded adolescents with physical conditions that compromised school attendance, making it impossible to respond directly to the data collection instrument.

Recruitment took place through visits to schools, and the vaccine card was requested, through the coordination, to verify the number of doses taken of the HPV vaccine. Subsequently, a communication was sent to parents and/or guardians, in order to explain the purpose of the research and request authorization to participate.

### Randomization and Blinding

We opted for randomization of the sample by clusters. The municipalities were divided into conglomerates with their school. The random allocation of clusters was defined by the simple random allocation process.

On that occasion, a lottery was used with numbered papers, sequentially, in recipients among the conglomerates, and then a random allocation was carried out to choose the schools. Thus, cluster A, municipality 2, was part of the IG, and cluster B, municipality 1, of the CG. It is worth noting that the schools were randomly selected to compose the Ig and the CG within the respective conglomerate. Moreover, adolescents were blind about the group they participated.

### Collection Instrument

The Knowledge, Attitude and Practice (KAP) survey used in the study was validated and pre-tested^(9)^. It is subdivided into seven sessions: 1. Personal data; 2. Sociodemographic, economic and cultural aspects; 3. Habits and health care; 4. Sexual and reproductive aspects; 5. Knowledge on HPV and its vaccination; 6. Attitude towards quadrivalent HPV vaccine; 7. Practice on quadrivalent HPV vaccine (exclusively completed by the researcher).

Its measurement took place as follows: a) Adequate knowledge: if participants have already heard about HPV and are able to inform at least one of these response alternatives about HPV: “It is a STI”, “Causes cervical/penis cancer/can turn into cancer”, or “Causes warts/disease/infection”; b) Inadequate knowledge: if participants had never heard about HPV or if they had heard about it, but could not say any of the information mentioned above about HPV; c) Appropriate attitude: if an adolescent mentions that she intends to be vaccinated; d) Inappropriate attitude: if an adolescent mentions that she does not intend to be vaccinated; e) Appropriate practice: if an adolescent receives the dose of the vaccine; f) Inappropriate practice: if an adolescent does not receive the vaccine dose.

### Data Collection

The pre-test data collection took place in August and September 2019. Standard and educational interventions took place in October and November 2019. Finally, the post-test was collected in December 2019 and January 2020.

First, CG adolescents were individually invited in the classroom to go to a private place at the school and respond to the pre-test of the KAP survey. After two months of standard intervention, these adolescents answered the post-test of the KAP survey via telephone, being asked again for information on the vaccination card about the doses of quadrivalent HPV vaccine to assess the outcome. Adolescents with whom there was no possibility of telephone contact or who did not respond to the requested instruments were discontinued from the study.

In the IG, the summoning of adolescents and the KAP survey pre-test application were carried out in the same way as in the CG. However, shortly after that moment, adolescents received the welcome message card. During the following two months, the educational intervention “Piss off, HPV!” was offered, in addition to the standard intervention.

Message card delivery was carried out by school professionals, collaborators who were blind as to the insertion group, to each adolescent, personally, in the classroom, twice a week, from Monday to Friday, on non-fixed days, in shifts. morning and afternoon, referring to the school’s opening hours, totaling 16 cards. At the end of this period, they received an acknowledgment message card for participating in the educational project.

The outcome considered was compliance with the quadrivalent HPV vaccine by adolescents, i.e., the administration of a vaccine dose proven in the vaccination card. The outcome assessment took place later with vaccination card checking at the time of carrying out the KAP survey post-test in person at school. Not responding to the KAP survey again or not taking the post-intervention vaccination card were considered study discontinuation criteria.

### Data Analysis and Treatment

Descriptive analysis was performed according to the frequency distribution of respondents’ responses. We performed the chi-square test to check homogeneity and compare the responses of individuals from the CG and IG, in order to find or not significant differences, in addition to comparing compliance with the quadrivalent HPV vaccine in relation to knowledge and attitude. The McNemar Test was used to analyze whether there was any change in the assessment of knowledge, attitude and practice before and after the intervention, in order to observe its effectiveness.

The practice assessment variable was used as the dependent variable, in which a regression model was used to analyze it. The statistical model used was the multiple logistic regression. The Hosmer and Lemeshow test was also carried out, for the adequacy of the model, and the stepwise variable selection method, to arrive at the final model.

The statistical significance level was set at 5% p < 0.05. The Statistical Package for the Social Sciences (SPSS), version 24.0, and software R were used for statistical data analysis.

### Ethical Aspects

The study was approved by the UFC Research Ethics Committee, under Opinion 2,645,679/2018, according to Resolution 466/2012 of the Brazilian National Health Council^(10)^. It is registered at the International Clinical Trials Registry Platform, under Universal Trial U1111-1254-5546 and Brazilian Clinical Trials Registry RBR-107hzdqt.

## RESULTS

The comparison of sociodemographic data and adolescents’ life habits found homogeneity, in general, between the groups (p < 0.05). With regard to age, the CG had a higher proportion of girls under 12 years of age (65.8%) than the IG (40.7%). Regarding race, the IG had a higher proportion of girls who considered themselves non-white (89%) than the CG (72.5%). As for religion, 47.5% of the CG considered themselves Catholic and 43.2% of the IG reported being Evangelical.

It is noteworthy that most participants in both groups had an income of up to US$363.63 (CG = 84.2%; IG = 68.6%), did not use cigarettes (CG = 99.2%; IG = 98.3%), alcohol (CG = 95%; IG = 94.1%) and illicit substances (CG = 100%; IG = 99.2%), used the public health service (CG = 88.3%; IG = 89.8%) and almost all adolescents (CG = 96.7%; IG = 96.6%) had not started their sexual life.

Of those who had started (CG = 3.3%; IG = 3.4%), the majority were over 12 years old (CG = 75%; IG = 100%) and did not manage (CG = 75%; IG = 100%). Of these, only one of the CG had become pregnant (25%), but spontaneously miscarried. None of them reported having an STI (100% in both groups), however 50% of the IG did not use any contraceptive method and 75% of the CG used condoms as the main method. Moreover, 75% of the CG and 50% of the IG did not perform gynecological prevention.

Table 1 shows that, before the intervention, knowledge was inadequate (p = 0.005) and attitude was adequate (p = 0.123) in most adolescents in both groups, although the IG initially presented less knowledge than the CG. It is also observed that adequate knowledge becomes greater in the IG after the application of the educational project (p = 0.000), surpassing the CG, despite having more initial knowledge.

**Table 1.** Comparison of knowledge, attitude and practice between groups before and after the educational intervention – Fortaleza, CE, Brazil, 2020.

Educational project “Piss off, HPV!”			Before		After	
Groups			CG^§^ (n = 120)	IG^‡^ (n = 118)	CG^§^ (n = 101)	IG^‡^ (n = 109)
**Knowledge**	**Adequate**	**N**	48	28	22	68
		**%**	40.0	23.7	21.8	62.4
	**Inadequate**	**N**	72	90	79	41
		**%**	60.0	76.3	78.2	37.6
	p–value*		**0.005**		**0.000**	
**Attitude**	**Adequate**	**N**	112	104	88	102
		**%**	93.3	88.1	87.1	93.6
	**Inadequate**	**N**	8	14	13	7
		**%**	6.7	11.9	12.9	6.4
	p–value*		0.123		0.087	
**Practice**	**Adequate**	**N**	**–**	**–**	24	89
		**%**	**–**	**–**	23.8	81.7
	**Inadequate**	**N**	**–**	**–**	77	20
		**%**	**–**	**–**	76.2	18.3
	p–value*		**–**		**0.000**	

* McNemar test; ^§^Control group; ^‡^Intervention group.

The questions assessed about knowledge (“Have you heard about HPV?”, “What do you think HPV is?” and “Have you heard about the quadrivalent HPV vaccine?”) were positively influenced post-intervention.

Regarding the post-intervention attitude, there was no significant difference when compared to the pre-test (p = 0.087). However, in the specific questions about thoughts about vaccines in general and whether they would take the HPV vaccine, there was an influence after the educational project implementation, becoming more effective in the IG (p < 0.005).

However, a relevant fact to be highlighted is that the CG showed a higher percentage of inadequate knowledge and attitude after the intervention, when compared to the IG.

Regarding the practice, it is highly associated with the groups, verifying that IG presented a frequency of practice assessed as adequate higher than the CG (p = 0.000), inferring that the intervention was significant.

Table 2 showed that compliance depends on the variables of knowledge and attitude, since adequate knowledge and attitude are associated with the compliance of adolescents with HPV vaccination (both p = 0.000).

**Table 2.** Association of compliance with quadrivalent HPV vaccine in relation to attitude and knowledge after the educational intervention – Fortaleza, CE, Brazil, 2020.

Educational project “Piss off, HPV!”	Compliance with the quadrivalent HPV vaccine				p-value^†^
	Yes (n = 113)		No (n = 97)		
	N	%	N	%
**Knowledge**					
Adequate	64	56.6	26	26.8	**0.000**
Inadequate	49	43.4	71	73.2	
**Attitude**					
Adequate	110	97.3	80	82.5	**0.000**
Inadequate	3	2.7	17	17.5	

† Chi-square test.

Also related to compliance with the quadrivalent HPV vaccine, Table 3 revealed that the variables age, knowledge and attitude explain approximately 70% of the practice. Age greater than or equal to 12 years is significant for the model at the level of 5% (p = 0.017; OR = 2.08), evidencing that adolescents aged 12 years or older are approximately twice as likely to have compliance to the quadrivalent HPV vaccine than those younger than 12 years.

**Table 3.** Variables related to the final model of logistic regression regarding quadrivalent HPV vaccine compliance – Fortaleza, CE, Brazil, 2020.

Final model variables		Coefficient	Standard error	Wald	DF	Sig.	Exp (B) Odds Ratio (OR)
**Step 9**	**Adequate post-intervention attitude**	1.707	0.666	6.560	1	**0.010**	5.510
	**Adequate post-intervention knowledge**	0.923	0.316	8.523	1	**0.004**	2.516
	**Age ≥ 12 years**	0.735	0.309	5.650	1	**0.017**	2.085
**Intercept**		–2.150	0.660	10.624	1	**0.001**	0.116

Adequate knowledge (p = 0.004) and attitude (p = 0.010) after intervention were also significant for the model, indicating that adolescents with adequate knowledge are 2.5 times more likely to comply with the quadrivalent HPV vaccine than those with inadequate knowledge (OR = 2.516) as well as adolescents with an adequate post-intervention attitude are 5.5 times more likely to be vaccinated (OR = 5.510).

## DISCUSSION

After the intervention, it was observed that adequate knowledge was greater in the IG, especially regarding hearing about HPV and its vaccine and what HPV is. The difference between the groups’ knowledge at the beginning of the research is also noteworthy, being lower in the IG, i.e., adolescents had less knowledge, and the result after the intervention could have been influenced by the higher percentage of inadequate initial knowledge. However, even with less initial knowledge, at the end of the intervention, they had adequate knowledge and much better than the CG, which reinforces the effectiveness of the intervention in terms of knowledge.

Corroborating with the present study, when asked about what HPV was in an experimental research with educational intervention, also carried out in Brazil, Rio Grande do Sul, the level of knowledge of school adolescents was also higher in the IG, showing a significant increase of 23.5% when compared to the CG (p < 0.000)^(11)^.

Internationally, a study with an educational session on HPV in Bamako, Mali, found that knowledge about the virus was very incipient and that only 3.4% of adolescents knew that HPV is an STI, and 7% of participants correctly stated that HPV is one of the main causes of CC before the educational session. Thus, participants’ knowledge increased significantly after the intervention, about 30%^(12)^. Another study, carried with Korean-American women, who also used written language as an educational strategy, showed significant differences in knowledge about HPV and post-intervention HPV vaccination^(13)^.

Differing from what was found in the present study, a research carried out in southeastern Brazil, with the aim of assessing an educational intervention on knowledge and attitude of adolescents about the vaccine, did not identify a statistically significant difference in terms of adequate knowledge. In the assessment of previous knowledge about HPV and its vaccine, it was observed that 8.5% of women knew about the topic, and, in the comparison of this item, there were also no differences (p > 0.05)^(9)^.

It appears that, in the studies described, knowledge may vary according to the population’s region and personal characteristics, such as age and education, as well as the context in which they are being assessed, i.e., public or private school, health unit, among others, and the moment, if it occurred soon after the intervention or weeks later.

When talking about attitude, it is observed, in the current research, that it was adequate in both pre- and post- intervention groups. However, thoughts about vaccines in general and whether to take the quadrivalent HPV vaccine were positively influenced after the intervention in the IG, resulting in an increase in the percentage of adequate attitude.

A study carried out in Malaysia showed that the number of schoolchildren who intended to be vaccinated against HPV was quite high (86.6%), and this desire to be vaccinated was significantly associated with knowledge about CC (p = 0.042)^(14)^. This vaccination-related attitude was also high among study participants in Mali; however, after the educational session, there was an increase in vaccine acceptance, especially among adolescents (from 75.3% to 91.8%)^(12)^.

The intervention through text message also presented significant increase statistical, intending that study participants receive the HPV vaccine (p < 0.001), with the percentage of participants who indicated an intention to receive the HPV vaccine within a one-year period increased from 63.3% to 96.7% (p = 0.009)^(13)^.

In the comparative analysis regarding the attitude towards the quadrivalent HPV vaccine in a Brazilian experimental study, the group submitted to the educational action showed the correct attitude in 37% of interviewees, against 23% of those who had not received intervention through the preliminary educational action (p = 0.044)^(9)^.

Research carried out with Italian pre-adolescents to assess knowledge, practices and attitudes regarding HPV infection and vaccination, showed that there is an overall positive attitude towards the importance of getting the vaccine and the severity of HPV-related illnesses even before educational sessions^(15)^, in line with data evidenced in this research.

It can also be seen that, according to the results presented in the present study, even adolescents who do not have adequate knowledge are willing to learn, as they seem to consider the subject as something important. Attitude is to interpret events according to certain predispositions. It is a trend, characterized as an intermediate variable between the situation and the response to that situation^(16)^, demonstrating how this public is open to new knowledge about the HPV vaccine and should be a constant target of health education in different contexts, such as family, school and health.

It is observed that educational interventions are capable of improving participants’ attitude towards HPV vaccination. However, it is important to emphasize that, from the beginning, there is already positive adolescents’ attitude towards the vaccine. This attitude, however, is not associated with adequate knowledge. Thus, it is believed that adequate information promotion, through effective educational strategies, significantly contributes to the acquisition of knowledge necessary for vaccine compliance.

In an Italian study, which assessed the possible association between the characteristics of young adults and the scores of knowledge and attitude, the variables significantly related to the highest scores were attending a health university, using social networks for up to two hours a day, history of STIs and HPV-related lesions, having heard about HPV prior to the survey, and reporting good economic status^(17)^.

Furthermore, family members and the mass media as a source of information about HPV are significantly related to worse knowledge scores. On the other hand, professionals specialized in the gynecological area are reported as a source of information, with significantly higher knowledge scores^(17)^.

Regarding the source of information about the quadrivalent HPV vaccine, individuals who reported teachers, information material and health professionals had significantly higher attitude scores^(17)^. Although there are non-modifiable factors, such as age, in the process of compliance with HPV vaccination, studies reinforce how fundamental the implementation of school and home education is, ensuring that effective information about the vaccine is shared among students and between adolescents and their families.

In addition, social networks can be allies in promoting sexual and reproductive health, as they reach a wide audience, including adolescents. Faced with the reality of using the virtual environment as a means of searching for information and communicating among peers, social networks need to be considered as a strategic environment for promoting adolescent health^(18)^. Thus, it is understood that there must be investment in the dissemination of clear, objective and attractive information to the adolescent public, in order to promote effective communication aimed at promoting health.

However, a finding evidenced in the present research is worrying, because the inadequate knowledge of the CG, which received standard guidelines from the school/health service, increased in the post-test, as well as the inadequate attitude, despite adolescents’ attitude have been generally adequate.

It is a fact that when adolescents are oriented at school by health professionals and teachers about HPV and the importance of vaccination, their knowledge, attitude and practice, they tend to become more effective^(19)^. However, such data raises doubts about the quality and the way in which these guidelines are being passed on in the current scenario.

According to a review, which aimed to describe the factors related to receptivity to the quadrivalent HPV vaccine, knowledge can act as a barrier or facilitator of this receptivity. Fear and increased refusal may be motivated by distorted information or insufficient secure data. In contrast, knowledge derived from science can be applied to educate individuals and increase receptivity. Another context, which generates permanent negative repercussions that are difficult to reverse, causing difficulty in receptivity to vaccines, is the dissemination of data without scientific evidence by social networks or by groups against vaccination^(20)^.

The school is a strategic place of orientation in health. It is also worth mentioning its fundamental role in promoting the sexual education of young people, as the school environment can be a place of relevant transformations in adolescents’ lives and health The integration between schools and health services needs to be implemented in order to address the health needs of students, generating empowerment of information and autonomy in their attitudes and practices for safe sexual initiation and STI prevention. However, for this to become effective, it requires understanding the family and its culture, since this understanding is considered a determining factor for health problems and the health education process^(21)^.

With regard to practice, the current survey results revealed that the educational intervention was significant for compliance with the quadrivalent HPV vaccine in the IG. The use of message cards as a means of sharing information resulted in the acquisition of adequate knowledge, and such knowledge, associated with the adequate attitude already presented by adolescents and strengthened by the intervention, seems to have influenced the decision in favor of the vaccine.

In a randomized study aimed at parents and adolescents aged 11 to 17 years, with the aim of encouraging HPV vaccination through a digital video, it was found that, between the intervention and control clinics, adolescents who participated in the intervention were almost twice as likely to receive a vaccine dose (OR = 1.82; p < 0.001)^(22)^.

Another study that tested the feasibility and effectiveness of a seven-day text message intervention to increase knowledge, attitude, and practice of HPV vaccination in 30 Korean-American women concluded that 16.7% of them reported having received the first HPV vaccine dose one week after completing the intervention program. Others, 13.3%, reported receiving the first dose of vaccine at the three-month follow-up visit, indicating that 30% of participants received the HPV vaccine after intervention^(13)^.

A Swedish study that aimed to promote HPV vaccination among students, in addition to increasing the use of condoms, found that face-to-face educational intervention in schools increased the likelihood that students would actually be vaccinated. The proportion of girls vaccinated in the IG was 52.5% before and 59% after the intervention, while no difference over three months was observed in the CG (60.9%) (p = 0.02)^(23)^.

In line with the data found in this research, scientific studies bring the association between HPV vaccination and educational intervention. When there is something that reinforces information, motivates participants and stimulates memory, the trend is for action, and this is the role of educational intervention, to empower individuals on the subject for positive decision-making.

A review carried out with the aim of investigating educational technologies constructed and/or used to promote HPV vaccination identified printed materials, such as flyers, messages from electronic devices, internet pages, computer programs, videos and radio soap operas. The development of these materials gave rise to creative, reliable and useful tools for health education, showing a positive impact on knowledge and compliance from their application with the target audience^(24)^.

Regardless of the type of educational technology used during an intervention, it is essential that this practice is encouraged in health education processes, in a participatory way, and that the target audience is an active agent in the promotion of their health, such as the intervention analyzed in the present study.

It is important to emphasize the school’s educational role, together with these health professionals, since vaccination practice is linked to this environment, confirmed in an Italian study, which investigated factors associated with the refusal of HPV vaccination in 141 young women. In this investigation, it was observed that one of the factors associated with not receiving the vaccine were lower participation in school seminars on HPV (OR = 0.25; p = 0.028) and lower perception of the benefits of vaccination (OR = 0.41; p = 0.044)^(6)^.

Undoubtedly, educational health technologies can be promising tools to promote health, given the satisfaction and increase in individuals’ knowledge, attitude and practice. Educational interventions using message cards as technology help in compliance with HPV vaccination, reducing the burden of CC and other types of associated neoplasms for younger populations, as it is simple, attractive and easy to read.

Despite being carried out with methodological rigor and showing valid results, the study had as main limitations: establishing effective communication via telephone with adolescents, but with the aim of mitigating losses; reinforce communication between school, parents and researchers for continuity of the research, in addition to other options for telephone contacts; punctually delivering the message cards in a relatively short period of time between the intervention and the post-test with vaccination card double-checking; and suggesting the development of further research to follow participants for a longer period of time, in order to assess the continuity of the educational intervention’s confirmed effectiveness.

As it is a low-cost technology that is simple to apply, it can be incorporated into the care process in different environments, such as schools, in periods that precede the vaccination campaign and health institutions in consultations and actions, as well as by Community Health Workers at home, being an important tool for building knowledge, not only of adolescents, but also of the family.

In nursing care, it can be a useful tool for the development of educational actions about HPV vaccination and to improve care at the primary level with regard to the prevention of pathologies associated with the virus.

## CONCLUSION

It is concluded that the educational project “Piss off, HPV”, using message cards, was effective for adolescents’ knowledge and compliance with the quadrivalent HPV vaccine. The variables age, knowledge and attitude explain approximately 70% of the practice. Adequate post-intervention knowledge and attitude, in addition to age greater than or equal to 12 years, increase the chance of compliance with the quadrivalent HPV vaccine.

Given the positive effects of educational intervention, it can be incorporated into the care process in different environments, as it is low-cost and simple to apply, such as in schools, especially in the periods before the vaccination campaign, such as in health institutions, by the team responsible for consultations and actions, as well as in households, being an important tool for building knowledge, not only for adolescents, but also for the family.
